# Synergistic larvicidal effects of temephos and *Bacillus thuringiensis israelensis* against *Aedes albopictus*: a laboratory and field study

**DOI:** 10.3389/fvets.2026.1930489

**Published:** 2026-10-20

**Authors:** Yuyan Wu, Qinmei Liu, Jimin Sun, Yifang Hong, Ying Fu, Dianying Zhou, Feng Ling, Guoqin Jiang

**Affiliations:** 1 Zhejiang Provincial Center for Disease Control and Prevention, Hangzhou, Zhejiang, China; 2 Shaoxing Center for Disease Control and Prevention, Shaoxing, Zhejiang, China; 3 Keqiao District Center for Disease Control and Prevention, Shaoxing, Zhejiang, China

**Keywords:** *Aedes albopictus*, *Bacillus thuringiensis*, drug resistance management, synergistic effect, temephos, vector control

## Abstract

**Background:**

*Aedes* mosquito-borne diseases, such as dengue fever, pose significant challenges to global public health. Mosquito management is regarded as one of the most critical measures to control these diseases. Temephos has been widely used for larval mosquito control, but its sustained application has resulted in the emergence of resistance in many regions in China. *Bacillus thuringiensis israelensis* (Bti) offers an environmentally friendly alternative, but its field efficacy against late-instar larvae is limited. Previous studies have demonstrated the synergistic potential of combining temephos and Bti. Therefore, this study aims to determine the most effective combination ratio through laboratory bioassays and to evaluate the practical efficacy of this mixed formulation under field conditions.

**Methods:**

Laboratory bioassays were conducted with early fourth-instar *Aedes albopictus* (Skuse, 1894) larvae to determine the co-toxicity coefficient (CTC) of 3 mixed formulations (temephos: Bti = 1:1, 1:3, and 1:5). In field trials, the mixed formulation with the greatest synergistic effect was used to evaluate its insecticidal effect under field conditions. A total of 150 natural breeding containers in one village in Shaoxing City were randomly selected and assigned to 3 groups (treatment group A, treatment group B, and the control group). Two application regimens (half-monthly application at twice the 99% lethal concentration [2 × LC99] vs. monthly application at 4 × LC99) were used in the treatment groups, and larval densities were monitored over a 3-month period to assess their larvicidal effects.

**Results:**

The CTC values for all three mixed formulations were 141.477, 140.299, and 150.009, respectively (all exceeding 120), and mixed formulation C (temephos: Bti = 1:5) was selected for field trials. Over the 3-month period, the standard rates of reduction in mosquito larval density were 100.00%, 100.00%, and 100.00% in group A, while group B showed reductions of 96.80%, 87.03%, and 96.79%. Both treatment groups exhibited significantly lower larval densities than the control group (*p* < 0.001), while no statistically significant difference was found between them.

**Conclusion:**

The combination of temephos and Bti showed a synergistic effect under laboratory conditions, and the mixed formulation demonstrated effective larvicidal activity under field conditions.

## 1 Introduction

*Aedes* mosquitoes are vectors for a diverse range of pathogens that can cause severe human diseases, such as dengue fever, Zika disease, chikungunya fever, and yellow fever. Hundreds of millions of cases are reported every year as a result of *Aedes* mosquito-borne virus infections, and the cumulative economic cost of these infections has reached $318 billion since 1975 (1). Among these diseases, dengue represents a particularly significant public health burden. Approximately 3.9 billion people are at risk for dengue fever, and it occurs in nearly 390 million people annually, resulting in 500,000 hospitalizations and 20,000 deaths (1–4). In China, more than 150,000 dengue cases have been reported over the past 20 years (2005–2025) (5). It is estimated that the normalized disability-adjusted life years from 2010 to 2019 in China attributable to dengue fever were 2.71 person-years per million population (6).

Mosquito-borne diseases are transmitted to humans through bites from mosquitoes infected with pathogens. The primary vector for dengue virus transmission in China, particularly in the southern and central provinces, is *Aedes albopictus* (the Asian tiger mosquito), a species highly adapted to peridomestic environments; however, in some areas, such as southern Yunnan Province, *Aedes aegypti* (Linnaeus, 1762) also plays a critical role in virus transmission (7). To control dengue fever in China, it is essential to manage *Aedes albopictus*. Chinese guidelines, such as the “Dengue Fever Prevention and Control Plan (2025 Edition),” mandate that when a local sporadic outbreak (non-imported) occurs, an urgent and aggressive vector control response should be implemented (8). A key operational target is to reduce the Breteau Index in areas where individuals with confirmed dengue infection have lived or worked to below 5 within 3 days of case confirmation. To achieve this target, public health authorities typically initiate a large-scale, intensive application of chemical insecticides to rapidly reduce both adult and larval mosquito populations.

Temephos is an organophosphate insecticide that has been used in larval mosquito management for decades. It is recommended by the World Health Organization (WHO) as the preferred larvicide because of its high efficacy against mosquito larvae, low toxicity to humans, livestock, and fish, and approval for use in potable water (9, 10). Consequently, temephos is the primary larvicide employed in both routine and emergency control of major vector species, including *Ae. albopictus*, *Culex pipiens pallens* Coquillett, 1898, and *Anopheles sinensis* Wiedemann, 1828. The sustained and intensive use of temephos has resulted in significant resistance in *Ae. albopictus* populations. A large number of previous studies, including those by Zou et al. (11, 12), Tu et al. (13), Zhang Yu et al., and Ma Ming et al., have revealed that *Ae. albopictus* populations developed resistance to temephos in Jiangsu, Chongqing, Shanghai, and Zhejiang, respectively (11–14). This emergence of resistance may reduce the efficacy of temephos in killing *Ae. albopictus* larvae, thereby hindering the timely containment of outbreaks. Therefore, there is an urgent need to identify a novel, evidence-based strategy for larvicide application that can ensure rapid vector suppression while simultaneously mitigating the further development of resistance.

*Bacillus thuringiensis* israelensis (Bti) is a spore-forming bacterium that produces protein crystals toxic to dipteran larvae, including mosquitoes and black flies (15, 16). As a biological larvicide, Bti offers several advantages over conventional chemical insecticides, including high target specificity, minimal environmental persistence, and low toxicity to non-target organisms, including humans and beneficial insects. Because Bti acts through complex interactions with receptors in the larval mosquito gut, the development of rapid resistance is considered less likely (15). Numerous studies have demonstrated the potent larvicidal activity and residual efficacy of Bti against *Aedes* mosquito larvae (17–21). However, field applications have also identified a limitation of Bti, with formulations often exhibiting reduced efficacy against late-instar, particularly fourth-instar, larvae compared with younger instars (22). To overcome the limitations of individual agents and potentially enhance overall efficacy, researchers have explored combining Bti with chemical insecticides. Li et al. demonstrated in 2017 that combining Bti with various chemical insecticides, including two pyrethroids and one organophosphate, resulted in significant synergistic larvicidal activity against *Cx. pipiens pallens* (23). Narkhede et al. found that Bti was most compatible with temephos after evaluating its compatibility with various larvicides (24). These findings suggest that Bti has the potential to interact synergistically with organophosphates such as temephos.

Given this background, there is an urgent need to develop a practical and effective strategy for controlling *Ae. albopictus* larvae under field conditions. The combined use of temephos and Bti may represent a promising strategy for achieving rapid larval mortality and improving overall efficacy, although the optimal combination ratio and larvicidal effect under field conditions were unknown. In this study, we investigated the potential of combining temephos and Bti. The aim of this study was to determine the most effective ratio using laboratory bioassays. The most promising formulation was then evaluated in a residential field setting to assess its efficacy under field conditions.

## 2 Materials and methods

### 2.1 Experimental bioassays

#### 2.1.1 Mosquito larvae

The *Ae. albopictus* mosquito larvae used in this study were obtained from a laboratory colony of a susceptible strain maintained at the Department of Vector Biology, Zhejiang Provincial Center for Disease Control and Prevention, China, and originally sourced from the Chinese Center for Disease Control and Prevention (25). The mosquito larvae were reared in dechlorinated water under controlled conditions following the Chinese Agricultural Industry Standard (NY/T 1964.3–2010), with a relative humidity of 75 ± 10% and a photoperiod of 14:10 (light:dark) h (26). All bioassays were conducted using early fourth-instar larvae.

#### 2.1.2 Chemicals tested

Temephos (94.69%) and Bti (7,000 international toxic units [ITU]/mg) were obtained from the Chinese Center for Disease Control and Prevention, China. The primary stock solutions of 200 mg/L Bti and 10 mg/L temephos were diluted using distilled water and acetone, respectively, to prepare the test concentrations and different combinations. Distilled water was used as the control. Three binary mixtures (A, B, and C) were prepared by mixing temephos and Bti at different mass ratios (1, 3, 5), based on the literature (23). The compositions of temephos, Bti, and their mixtures are listed in Table 1. The mixtures were homogenized and stored at 4 °C until use.

**Table 1:** Composition of temephos, Bti, and their binary mixtures.

Formulation	Mass ratio (temephos: Bti)	Temephos content (g/g mixture)	Bti content (g/g mixture)
Temephos	1:0	1	0
Bti	0:1	0	1
Mixed formulation A	1:1	0.5	0.5
Mixed formulation B	1:3	0.25	0.75
Mixed formulation C	1:5	0.17	0.83

Bti, Bacillus thuringiensis israelensis.

#### 2.1.3 Larval bioassays

All bioassays were conducted at a room temperature of 26 ± 1 °C and a relative humidity of 70 ± 5%. Larvicidal activity was evaluated for individual temephos and Bti treatments and mixed formulations A–C using the larval immersion method described in the Chinese national standard “Sensitive Baseline Method for Determining Median Lethal Concentration” (GB/T 26347–2010) (27). A total of 30 larvae in 50 mL of water were placed in 150 mL of test medium containing a particular concentration of the tested insecticide. All bioassays were performed in triplicate for each concentration, and mortality was recorded after 24 h of exposure. Larval mortality in all assays was recorded and calculated using the Abbott formula (Abbott, 1925). The toxicity regression equation and median lethal concentration (LC50) values for each formulation were calculated to determine the co-toxicity coefficient (CTC). The combination with the highest CTC (defined as mixed formulation X) was regarded as the most effective and was subsequently used in field trials.

### 2.2 Field trials

Field trials were conducted in Shaoxing City, Zhejiang Province, China, in 2024 by professional staff from the centers for disease control and prevention (CDC) in Shaoxing and Keqiao. These trials were conducted from early July to late September, when the temperature ranged from 26 °C to 31 °C and rainfall ranged from 147.7 to 264.6 mm. One village in Shaoxing City was selected as the study site. A total of 150 large- or medium-sized water containers (capacity ≥ 4 L and ≤ 25 L), including flowerpots, earthenware jars, and cisterns, that permanently held standing water and were naturally infested with *Ae. albopictus* larvae were randomly selected within the village. The containers were allocated to three groups based on their spatial distribution. Fifty containers were designated as Treatment Group A. Within a radius of 5 m of each container in Group A, an additional 50 containers meeting the same inclusion criteria (permanent standing water and presence of *Ae. albopictus* larvae) were selected and designated as Treatment Group B. An additional 50 containers located in close proximity to both treatment groups were selected as the Control Group. The containers in the 3 groups were located in close proximity because larval density might be affected by mosquito movement and oviposition patterns in the surrounding area. After the containers were selected, graduation lines were marked on each container according to its size, corresponding to volumes of 5, 10, and 20 L. At each time point, excess water was either removed or clear pond water was added to bring the water level to the appropriate graduation mark, thereby standardizing the water volume in each container and allowing accurate dosing of formulation X. A primary stock solution of formulation X (200 mg/L) was prepared using distilled water in the laboratory for on-site use.

Treatment Group A: Formulation X was applied once every 2 weeks at a concentration equivalent to twice the laboratory-derived 99% lethal concentration (2 × LC99) by adding 5.50, 11.00, and 22.00 mL of the standard solution (200 mg/L of formulation X) to containers containing 5, 10, and 20 L of water, respectively.

Treatment Group B: Formulation X was applied once a month at a concentration equivalent to four times the laboratory-derived 99% lethal concentration (4 × LC99) by adding 11.00, 22.00, and 44.00 mL of the standard solution (200 mg/L of formulation X) to containers containing 5, 10, and 20 L of water, respectively.

Control Group: No treatment was administered during the study period (Figure 1).

**Figure 1:**
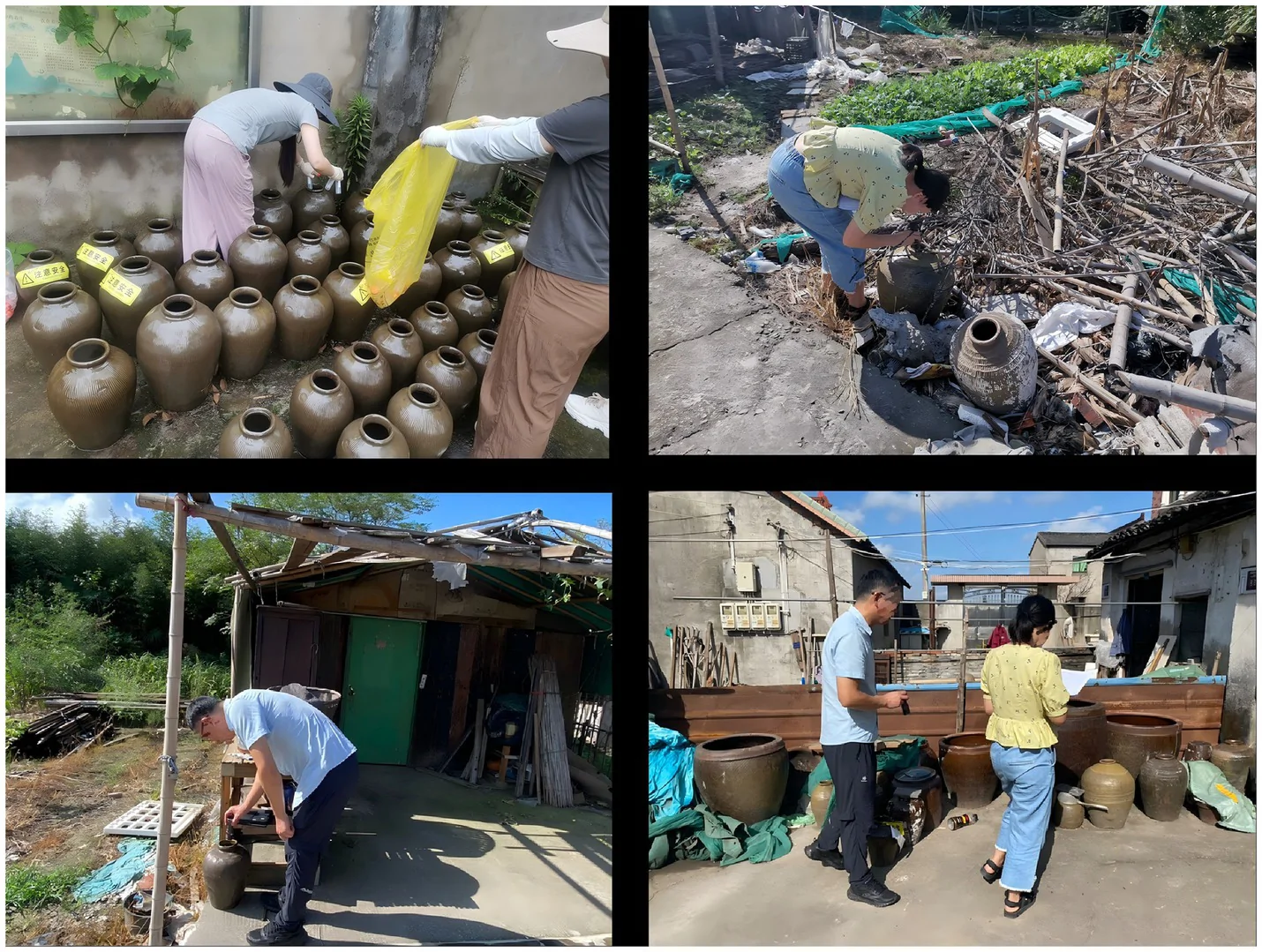
Field personnel administering chemicals to containers in the different groups.

Before the trials began, larval density was monitored to determine whether the baseline mosquito density was comparable among the three groups. The trial period was divided into 3 consecutive 1-month cycles from July 1 to September 29. To assess the larvicidal efficacy of mixed formulation X, larval density was monitored at 1, 3, 7, 15, and 30 days after each application. The larval density was determined by counting all mosquito larvae in each container at each monitoring time point; the larvae were returned to the containers after counting. The percentage reduction in larval density was then calculated.

### 2.3 Statistical analysis

Statistical analyses were performed using IBM SPSS Statistics, version 23.0.

Experimental bioassays: Corrected larval mortality data were subjected to log-probit analysis, and the LC50 values were calculated. The CTC was calculated using the method described by Tang (28). According to the classification by Tan et al. (29), the joint action of insecticides was categorized as antagonistic when CTC < 80, additive when CTC was between 80 and 120, and synergistic when CTC > 120.

Field trials: Non-parametric tests for multiple independent samples were used to compare larval mosquito densities among the three groups (groups A and B and the control group) before the trials began. Generalized linear mixed models with negative binomial regression were used to evaluate differences in larval mosquito density among the groups during the trials. Larval mosquito density was the dependent variable, with group, average temperature, and rainfall during the week preceding each monitoring time point (data from the China Meteorological Administration, CMA) included as fixed effects and date included as a random effect. Means and standard errors were estimated using generalized linear mixed models. Statistical significance was set at *p* < 0.05.

Larval density = number of mosquito larvae/number of containers.

Rate of decrease in mosquito larval density = [(larval density before treatment − larval density after treatment)/larval density before treatment] × 100%.

Standard rate of decrease in mosquito larval density = [(rate of decrease in mosquito larval density in the treatment group−rate of decrease in mosquito larval density in the control group)/(1−rate of decrease in mosquito larval density in the control group)] × 100%.

## 3 Results

### 3.1 Larvicidal activity of various insecticides in experimental settings

The control mortality percentages for each bioassay were 0%. The regression equations and LC50 values were calculated using 24-h mortality data for 2 individual insecticides (temephos and Bti) and 3 mixed formulations (A, B, and C; Table 2). The CTCs of the 3 mixed formulations were 141.477, 140.299, and 150.009, respectively (all exceeding 120), and all 3 mixed formulations showed synergistic effects. Mixed formulation C showed the highest CTC value, indicating the greatest synergistic potency, and was selected as the candidate formulation for subsequent field trials.

**Table 2:** Larvicidal effects of temephos, Bti, and their combinations against *Ae. albopictus* larvae.

Insecticides	Regression equation	LC50 (95% CI, mg/L)	CTC (95% CI)	Effect
Temephos	Y = 8.7535 + 4.9335X	0.017 (0.010, 0.022)	/	/
Bti	Y = −3.7161 + 4.1003X	8.059 (6.901, 9.249)	/	/
Mixed formulation A	Y = 11.8462 + 7.2870	0.024 (0.021, 0.026)	141.477 (133.204, 150.634)	Synergistic
Mixed formulation B	Y = 10.3757 + 7.8445X	0.048 (0.044, 0.052)	140.299 (135.281, 145.907)	Synergistic
Mixed formulation C	Y = 12.2929 + 10.4414X	0.067 (0.064, 0.069)	150.009 (145.149, 155.041)	Synergistic

Bti, Bacillus thuringiensis israelensis; CI, confidence interval; CTC, co-toxicity coefficient; LC50, median lethal concentration.

### 3.2 Larvicidal effects under field conditions

#### 3.2.1 General information on field trials

Field trials were conducted in Yingjia Village, Shaoxing City, China. Before the trials, the larval density was assessed in the 3 groups, with mean larval densities of 6.94 (interquartile range [IQR]: 4.50–10.00), 6.29 (IQR: 2.00–6.00), and 7.12 (IQR: 2.50–10.00), respectively. No statistically significant differences were observed among the 3 groups before the trials (*F* = 0.069, *p* = 0.933 > 0.05; Table 3).

**Table 3:** Mosquito larval density in different groups before and after the trials.

Date			Control group		Treatment group A			Treatment group B		
			Larval density	Rate of decrease in mosquito larval density	Days after insecticide application	Larval density	Rate of decrease in mosquito larval density/standard rate of decrease in mosquito larval density	Days after insecticide application	Larval density	Rate of decrease in mosquito larval density/standard rate of decrease in mosquito larval density
Trial initiation	July 1 (Larval density observed before the trials)		6.94	0%	0 (First insecticide application after observation)	6.29	0%	0 (First insecticide application after observation)	7.12	0%
After the trials	First month	July 2	6.90	−22.78%	1	0	100%/100%	1	0	96.07%/96.80%
		July 4	6.96		3	0		3	0	
		July 8	5.34		7	0		7	0	
		July 16	16.28		15 (Second insecticide application after larval density observation)	0		15	0	
		July 17	13.30		1	0		No monitoring planned	None	
		July 19	10.36		3	0		No monitoring planned	None	
		July 23	3.34		7	0		No monitoring planned	None	
		July 31	5.70		15 (Third insecticide application after observation)	0		30 (Second insecticide application after observation)	0.28	
	Second month	August 1	4.14	37.19%	1	0	100%/100%	1	0	91.85%/87.03%
		August 3	1.22		3	0		3	0	
		August 7	2.24		7	0		7	0	
		August 15	7.84		15 (Fourth insecticide application after observation)	0		15	0	


		August 16	3.04		1	0		No monitoring planned	None	
		August 18	7.38		3	0		No monitoring planned	None	
		August 22	5.22		7	0		No monitoring planned	None	
	August 30	3.80	15 (Fifth insecticide application after observation)		0	30 (Third insecticide application after observation)		0.58		
	Third month	August 31	1.90	−31.21%	1	0	100%/100%	1	0	95.79%/96.79%
		September 2	3.78		3	0		3	0	
		September 6	4.40		7	0		7	0	
		September 14	16.16		15 (Sixth insecticide application after observation)	0		15	0	
		September 15	11.50		1	0		No monitoring planned	None	
		September 17	7.88		3	0		No monitoring planned	None	
September 21		11.22	7		0	No monitoring planned		None		
September 29		16.02	15		0	30		0.30		

After the trials, the mean larval densities over the 3-month period were 0, 0, and 0 in Treatment Group A; 0.28, 0.58, and 0.30 in Treatment Group B; and 8.52, 4.36, and 9.11 in the Control Group (Table 3).

#### 3.2.2 Larvicidal effects of different application modes of mixed formulation C

After the trials, natural fluctuations in larval mosquito densities were observed in the control group, with mosquito densities decreasing by −22.78%, 37.19%, and −31.21%, respectively, over the 3-month period compared with those before the trials (Table 3), suggesting that seasonal fluctuations in mosquito breeding persisted. Larval mosquito densities decreased by 100%, 100%, and 100%, with standard decrease rates of 100%, 100%, and 100%, respectively, after adjustment for the control group in Treatment Group A. In Treatment Group B, larval mosquito densities decreased by 96.07%, 91.85%, and 95.79%, with standard decrease rates of 96.80%, 87.03%, and 96.79%, respectively, after adjustment for the control group. Groups A and B exhibited significantly lower larval densities than the Control Group (*p* < 0.001 for both), whereas no significant difference in density was observed between Groups A and B (*p* > 0.05; Table 4).

**Table 4:** Differences in monitored mosquito density among groups.

Group	*t*-value	*p*-value	95% confidence interval (95% CI)	
			Lower	Upper
Group A *vs.* control group	−9.485	<0.001	−8.886	−5.774
Group B *vs.* control group	−9.235	<0.001	−8.692	−5.581
Group A *vs.* group B	−0.250	0.804	−1.749	1.362

CI, confidence interval.

## 4 Discussion

Vector management is crucial for controlling mosquito-borne diseases. However, increasing insecticide resistance has become a major challenge, making vector control more difficult (30, 31). According to previous monitoring data from Zhejiang Province (2020), the wild population of *Ae. albopictus* in Shaoxing had developed resistance to temephos, with a 12-fold resistance ratio greater than that of the susceptible strain. The results of the field trials in both groups showed a significant decrease in mosquito larval density at each monitoring time point, suggesting that the temephos–Bti mixture may retain larvicidal activity against mosquito populations that have developed resistance to temephos. However, the results were derived from a 3-month observation period, and the potential effects of this mixture on long-term resistance management require further investigation.

According to a previous study by Yuwei *et al*. (17) in 2018, the larval mosquito mortality rates at 1 and 2 weeks using Bti alone at a concentration of 2 × LC99 were <99.8% and <66.0%, respectively, under laboratory conditions. In 2015, Li Chun-xiao et al. (32) used 1% temephos granules at a dosage of 5 g/m^2^ to kill mosquito larvae in sewers under field conditions and found that 50% of the sewers had been re-infested with larvae after 2 weeks. In 2016, the World Health Organization also recommended the use of temephos to control mosquito larvae in open water bodies, with a treatment interval of 7–10 days (9). These findings indicate that the effective durations of both Bti and temephos for controlling mosquito larvae at the recommended dosage were generally less than 2 weeks. However, in this study, after combining Bti and temephos in appropriate proportions (mixed formulation C), the larvicidal effect under field conditions persisted for up to 1 month. In addition, the amounts of temephos and Bti in mixed formulation C were substantially lower than those used for the individual insecticides, with the amount of temephos at the LC99 level constituting 65.97% of that in pure temephos. This combination may therefore enhance larvicidal efficacy while reducing environmental contamination.

In this study, seasonal fluctuations in mosquito breeding activity were observed in the control group, with larval density increasing relative to the baseline during months 1 and 3 (−22.78% and −31.21%, respectively), which is consistent with the known seasonal peak of mosquito breeding in Shaoxing during July–September (33). The standard rate of decrease was used to adjust for these fluctuations, providing a conservative estimate of the actual decrease in larval density when the control density increased. In the field trials, we explored 2 insecticide application patterns with the aim of developing a validated application pattern for controlling *Aedes* mosquito-borne diseases that could rapidly and effectively reduce mosquito larval density. According to the results, in Group A, the standard decrease in larval mosquito density was 100% at each monitoring point throughout the 3-month period. In Group B, the standard decrease in larval mosquito density ranged from 87.03 to 96.80%. No statistically significant differences in larval mosquito density were observed between the 2 treatment groups. Theoretically, assuming that the container volume and water turnover were constant in the 2 groups, the dosages of the 2 insecticides applied within a month would be the same in the 2 application patterns; therefore, their environmental impacts would be expected to be similar. However, the labor requirements for the 2 application patterns differed, with the labor input for Group A theoretically being twice that for Group B. In practical applications, Group A could be adopted when more thorough larval control is required, whereas Group B could be chosen when lower labor input is desired.

A limitation of this study is that only 3 mixed proportions of temephos and Bti (ranging from 1:1 to 1:5) were tested in the laboratory experiments, and the possibility that higher Bti proportions might further enhance efficacy cannot be excluded. Although the field trials demonstrated effective larval control and the standard decrease in larval mosquito density consistently reached 100% in Group A, further research is required to evaluate combinations with other proportions and determine whether they provide additional benefits. Second, the field population in Shaoxing City showed resistance to temephos in 2020, but resistance was not directly assessed in the present study. Therefore, the efficacy of the mixed formulations in resistant populations requires further investigation. Third, although a 100% reduction in Group A was observed at each sampling time point, this finding should be interpreted with caution because it might have been influenced by temporary suppression of larval development, mortality at another life stage, oviposition avoidance following treatment, or insufficient sampling intensity rather than complete eradication of mosquito breeding. Finally, during the application process, we observed that water volumes in the containers could not be maintained precisely at the premarked graduation lines owing to evaporation and precipitation, resulting in fluctuations in the active ingredient concentrations relative to the target levels. Although such variability may affect the precision of concentration control, it reflects the actual conditions under which vector control interventions are implemented.

## 5 Conclusion

The combination of temephos and Bti showed synergistic effects under laboratory conditions, resulting in enhanced toxicity against *Aedes* mosquito larvae. The mixed formulation also demonstrated effective larvicidal activity under field conditions. These findings suggest that combining temephos and Bti may provide an effective strategy for larval mosquito control while allowing the use of lower amounts of the individual insecticides. Further studies are needed to evaluate the long-term effects of the combination on insecticide resistance and its environmental impact.

## Data Availability

The original contributions presented in the study are included in the article/supplementary material, further inquiries can be directed to the corresponding authors.
